# Vaccines in Children with Inflammatory Bowel Disease: Brief Review

**DOI:** 10.3390/vaccines9050487

**Published:** 2021-05-11

**Authors:** Susanna Esposito, Giulia Antoniol, Marialuisa Labate, Lucrezia Passadore, Patrizia Alvisi, Valeria Daccò, Chiara Ghizzi, Carla Colombo, Nicola Principi

**Affiliations:** 1 Paediatric Clinic, University Hospital, Department of Medicine and Surgery, University of Parma, 43126 Parma, Italy; giulia.antoniol@gmail.com (G.A.); marialuisa.labate@libero.it (M.L.); lucre.passadore@gmail.com (L.P.); 2 Pediatric Gastroenterology Unit, Maggiore Hospital, 40133 Bologna, Italy; patrizia.alvisi@ausl.bo.it; 3 Cystic Fibrosis Centre, Fondazione IRCCS Ca’ Granda Ospedale Maggiore Policlinico, Department of Pathophysiology and Transplantation, Università degli Studi di Milano, 20122 Milan, Italy; valeria.dacco@policlinico.mi.it (V.D.); carla.colombo@unimi.it (C.C.); 4 Pediatric Unit, Maggiore Hospital, 40133 Bologna, Italy; chiara.ghizzi@ausl.bo.it; 5 Università degli Studi di Milano, 20122 Milan, Italy; nicola.principi@unimi.it

**Keywords:** Crohn’s disease, inflammatory bowel diseases, ulcerative colitis, vaccination, vaccine

## Abstract

Incidence of inflammatory bowel diseases (IBDs), including Crohn’s disease (CD) and ulcerative colitis (UC), is increasing worldwide. Children with IBDs have a dysfunctional immune system and they are frequently treated with immunomodulating drugs and biological therapy, which significantly impair immune system functions and lead to an increased risk of infections. Vaccines are essential to prevent at least part of these infections and this explains why strict compliance to the immunization guidelines specifically prepared for IBD patients is strongly recommended. However, several factors might lead to insufficient immunization. In this paper, present knowledge on the use of vaccines in children with IBDs is discussed. Literature review showed that despite a lack of detailed quantification of the risk of infections in children with IBDs, these children might have infections more frequently than age-matched healthy subjects, and at least in some cases, these infections might be even more severe. Fortunately, most of these infections could be prevented when recommended schedules of immunization are carefully followed. Vaccines given to children with IBDs generally have adequate immunogenicity and safety. Attention must be paid to live attenuated vaccines that can be administered only to children without or with mild immune system function impairment. Vaccination of their caregivers is also recommended. Unfortunately, compliance to these recommendations is generally low and multidisciplinary educational programs to improve vaccination coverage must be planned, in order to protect children with IBD from vaccine-preventable diseases.

## 1. Background

Inflammatory bowel diseases (IBDs), including Crohn’s disease (CD) and ulcerative colitis (UC), are a group of immune-mediated chronic conditions that are presumed to occur in genetically susceptible subjects due to a dysregulated intestinal immune response to a number of environmental factors [1]. Among these, a decrease in the overall diversity of gut microbiota composition (i.e., dysbiosis) seems to play a major role [2,3]. IBD is thought to have a strong genetic component, since family history of IBD is among the greatest risk factor for the disease at all ages. Large genome-wide association studies of IBD found more than 200 common loci associated with the disease [1].

Pediatricians are often involved in the management of IBD cases as peak onset time of these diseases is adolescence, and among cases that develop under the age of 18 years, 4% occur under 5 years and 18% under 10 years [4]. Moreover, incidence of pediatric IBDs is increasing worldwide, even in traditionally low-prevalence regions, such as the Middle East and Asia. In the USA, the pediatric prevalence of IBDs overall increased from 33.0/100,000 in 2007 to 77.0/100,000 in 2016 (133%) [5]. In Singapore, mean annual incidence of pediatric IBDs was 0.23 per 100,000 from 1994 to 2004, and was almost 10-fold higher (2.28 per 100,000) during the following decade (2005–2015) [6]. Finally, cases of pediatric IBDs are difficult to be manage and accurate monitoring of clinical conditions of young patients for treatment adjustment is continuously needed. 

Infections are common problems that pediatricians following children with IBD must continuously monitor and eventually treat. Children with IBDs have a dysfunctional immune system. Moreover, in order to reduce clinical manifestation of IBDs, they are frequently treated with immunomodulating drugs and biological therapy [7], which significantly impair immune system functions and lead to an increased risk of infections [8,9,10]. Vaccines are essential to prevent at least part of these infections, and this explains why strict compliance to the immunization guidelines specifically prepared for IBD patients is strongly recommended by health authorities worldwide. However, as evidenced in adults with IBDs [11], even in children, several factors might lead to insufficient immunization. To reduce this risk, a specific immunization schedule must be followed. In this paper, present knowledge on use of vaccine in children with IBDs is discussed.

## 2. Immune System Function in Children with Inflammatory Bowel Disease (IBD) 

### 2.1. General Characteristics of Immune System in Children with IBD

IBDs are disorders generally driven by multiple genetic polymorphisms that are frequently common with other complex autoimmune and immunodeficiency diseases, such as ankylosing spondylitis and psoriasis, which can often present as extra-intestinal manifestations of IBDs. A total of 201 loci associated with IBDs were identified [12]. Their expression is strictly related to the intestinal barrier permeability and function and with all mechanisms that activate detection of pathogens by the innate immune system; and the following tolerogenic, inflammatory, and restitutive responses, which are essential for adaptive immunity development [13]. Genetic variants of IBD-specific loci found in IBD patients can impair all these functions, leading to abnormal immune response with increased risk of infection and poor response to antigen stimulation, including vaccine administration [14]. Several studies evidenced that the supposed alterations of the immune system functions potentially related to genetic variants are present in IBD patients. A disturbance of the cytokine profile in favor of pro-inflammatory cytokine overproduction was repeatedly reported [15,16,17,18]. CD is often described as a prototype of T-helper (Th) 1-mediated diseases with increased levels of tumor necrosis factor-alpha (TNF-a), interferon gamma (IFN-γ), and interleukin (IL)-12 and IL-1 in both the intestinal tissue and peripheral blood. UC is usually viewed as a Th2-type condition because of the increased intestinal expression of excessive Th2 response, with upregulated secretion of IL-5, IL-4, IL-10, and IL-13 [15]. In both cases, overexpression of pro-inflammatory cytokines was found to be associated with the initiation and progression of IBDs [16]. Moreover, circulating natural killer (NK) cells of IBD patients have an unbalanced metabolic profile, with faulty mitochondria and a reduced capacity to kill [17]. In addition, cases of IBD that develop in the first year of life and share common genes implicated in the development of primary immunodeficiencies have abnormal levels of circulating T-cells or specific T-cell subsets, such as T-helper (Th) 17 cells or regulatory T-cells (Tregs) [17]. As recent data show that cytokines produced by Th17 initiate and amplify the local inflammatory signs and promote the activation of counter-regulatory mechanisms targeting intestinal epithelium cells, it seems clear that genetic variants can significantly modify immune response [18]. Furthermore, IBD patients are at higher risk of infections related to malnutrition, which might involve up to 40% of children at the time of diagnosis [4]. It is also known that any infection might trigger a flare of IBD [4]. Therefore, protecting this group of patients against infections is of upmost importance.

### 2.2. Immune System Functions during IBD Course

As in adults, to control clinical manifestations of pediatric IBDs, some immunosuppressive drugs and biologics are prescribed. Among immunosuppressive drugs, corticosteroids (CS), thiopurines (azathioprine (AZA), cyclosporine, 6-mercaptopurine (6-MP)), and methotrexate (MTX) are the most frequently used. Among biologics, anti-TNF-α agents such as infliximab (IFX), adalimumab (ADA), and more recently, in selected off label cases—vedolizumab and ustekinumab—are those more commonly administered [19]. However, as childhood-onset IBDs are generally more aggressive and rapidly progressive than those diagnosed in adults [20,21], children are more frequently treated with CS and AZA/6-MP from the early stages of disease. Moreover, as many cases do not adequately respond and become steroid-dependent, use of TNF-α agents alone or in combination with immunomodulators is prescribed significantly earlier than in adults [22]. Although with different mechanisms, all these drugs can compromise immune system functions. Consequently, risk of impaired immune system functions is extremely common in children with IBDs shortly after the disease diagnosis [23]. The degree of immunosuppression that is considered high level and might hamper immune response or compromise safety includes patients receiving CS therapy with prednisone daily dose of ≥20 mg (or in patients weighing <10 kg, 2 mg/kg/day), MTX > 0.4 mg/kg/wk, AZA > 3 mg/kg/day, or 6-MP 1.5 mg/kg/day. Administration of anti-TNF-a agents at any dosage is sufficient to cause immunodepression [24].

## 3. Occurrence of Infections in Children with Inflammatory Bowel Disease (IBD) 

Although with exception, most studies that evaluated the risk of infections in adults with IBDs showed that it increased. A recent analysis of the long-life prevalence of relevant infections in a cohort of 6914 patients showed that it was globally 3% and 5% in immunosuppressed subjects [25]. Respiratory, intestinal and urinary infections were the most common. Opportunistic infections such as tuberculosis (TB) and herpes zoster (HZ) were also evidenced, with a prevalence of 2.6/1000 and 3.9/1000, respectively. TB was mainly related to the use of anti TNF-α preparations, whereas HZ with the administration of thiopurines alone or in combination with biologics. Infection-related mortality was 2.8% [25]. Similar findings were previously reported by Toruner et al. [26]. Administration of CS was found to be associated with an odds ratio (OR) for opportunistic infections of 3.4 (95% confidence interval [CI], 1.8–6.2), AZA/6-MP with an OR of 3.1 (95% CI, 1.7–5.5), and IFX with an OR of 4.4 (95% CI, 1.2–17.1). Use of combinations increased dramatically, with the OR reaching a value of 14.5 (95% CI, 4.9–43) [26].

As pediatric patients with IBD more frequently receive immunosuppressive therapy or biologics, it should be expected that children have a greater risk of infections than adults [20]. Results of pediatric studies do not support this hypothesis. Unfortunately, studies are few and the results are conflicting. Variations in study design, lack of uniformity in case definition, use of different IBD therapy, presence of comorbidities, and lack of long-term evaluation might explain the differences. Definitive conclusions cannot be drawn. However, in most cases, an increased risk was reported, and as in adults, a strict relationship with therapy was demonstrated. Invasive bacterial infections were relatively uncommon. The systematic review of the studies [27,28,29,30,31,32,33,34,35,36,37,38,39,40,41,42,43,44,45,46,47,48,49,50,51,52,53,54,55,56,57,58,59,60,61,62,63,64,65] carried out in children with IBDs treated with TNF-α agents published between 2000 and 2012 concluded that infection incidence was only slightly higher than that usually found in healthy subjects and that most infections were mild and in the upper respiratory tract. The incidence of mild infections ranged from 3% (1/38) to 77% (46/60), and from 0% (0/66) to 10% (6/60) for serious infections. Sepsis, gastrointestinal, and soft tissue infections were the most common severe infections [66]. Comparison of incidence of infections according to the type of therapy was made in a systematic review of 65 pediatric studies published before 2014, including a total of 5528 patients with 9516 patient-years of follow-up [67]. Global incidence of severe infections was lower in children than in adults, given the pharmacological agents, as there were 352/10,000 patient-years of follow-up (PYF), as compared to 654/10,000 PYF (incidence ratio [IR], 0.54; 95% CI, 0.43–0.67). Risk of infections with TNF-α agents was quite similar to that due to immunosuppressive drugs (333/10,000 PYF) and was lower than that due to CS (730/10,000 PYF; IR, 0.48; 95% CI, 0.40–0.58). Although some studies did not find a role of TNF-α inhibitors in favoring infection development in IBD patients [68], importance of biologics was further demonstrated when the data collected with the pharmaco-epidemiological and registry studies of pediatric IBDs were considered. IFX and ADA administrations were associated with serious infections in 8% of cases [45]. Moreover, in treated children, a risk of severe infections was found to be 5 times higher than that in patients without biologics [69]. However, when IFX and ADA were considered separately, the risk was totally ascribed to IFX, as children receiving ADA were not different from untreated patients [52]. 

Regarding vaccine-preventable diseases, role of IBD therapy on infection incidence and severity was mainly studied in adults, as evaluation in children is made difficult by the administration of vaccines before IBD development, in most cases. In general, all these infections were found to be more common and severe in patients, as compared to the controls, and use of immunomodulators or biologics further increased the risk. Use of biologics in adults with IBDs was associated with reactivation of HBV infection [70,71], although no case was described in children. 

Varicella and herpes zoster in IBD children was found to be associated with increased risk of hospitalization. The association was strong for both CD and UC, although greater for CD (varicella: OR, 12.75; 95% CI, 8.30–19.59, zoster OR, 7.91; 95% CI, 5.60–11.18 vs. varicella OR 4.25; 95% CI 1.98–9.12, zoster OR 3.90; 95% CI 1.98–7.67) [72]. Moreover, infection with VZV in patients treated with CS, thiopurine, and biologics could lead to the development of severe disseminated and occasionally fatal disease [73,74,75].

It is highly likely that *Streptococcus pneumoniae* infections are more common in IBD patients than in controls and that therapy might play a role in this regard. In a retrospective cohort and a nested case-control study, it was evidenced that the risk of pneumonia was higher in IBD adults than in non-IBD case (OR, 1.82; 95% CI, 1.75–1.88). Use of biologic medications (OR, 1.32; 95% CI, 1.11–1.57), CSs (OR, 1.91; 95% CI, 1.72–2.12), and thiopurines (OR, 1.13; 95% CI, 1.00–1.27) was independently associated with pneumonia [76].

Influenza is generally more severe in patients with a weakened immune system, including immunosuppression caused by medications. Regarding IBD patients, a retrospective cohort study including a total of 140,480 subjects revealed that these patients had an increased influenza risk (OR, 1.54; 95% CI, 1.49–1.63) and could suffer from a more severe disease (rate of hospitalizations 5.4% vs. 1.85%; *p* < 0.001) [77]. Systemic CSs were found to be independently associated with influenza occurrence (OR, 1.22; 95% CI, 1.08–1.38). 

Considering vaccine-preventable diseases overall, accordingly to what was reported for adults [10], it seemed likely that they have an increased risk of all these diseases, if the protection offered by vaccines is not achieved or is prematurely lost. This strongly influences the vaccination strategies recommended for these patients. 

## 4. Immunization of Children with Inflammatory Bowel Disease (IBD)

### 4.1. Compliance with Recommendations

Although most countries have detailed recommendations for immunization of children with IBD, several studies showed that the incidence rate of incomplete immunization schedule was significantly higher in IBD patients than in healthy controls [77,78,79,80]. A French study carried out in outpatients followed in six tertiary centers and five local hospitals from May to November 2011 showed that only 24% and 4% of children with IBD were vaccinated in agreement with the national immunization schedule and the recommendations for IBD, respectively [81]. Coverage was 87% for diphtheria–tetanus–poliomyelitis, 38% for hepatitis B (HB), 32% for pneumococcus, and 22% for influenza [81]. These findings were recently confirmed by a multicenter, retrospective cohort investigation including 430 children with IBD enrolled in 13 European centers, between July 2016 and July 2017 [82]. This study showed that vaccination rates at diagnosis were unsatisfactory for measles, mumps, and rubella (MMR) (89.3%), *Haemophilus influenzae* type b (Hib) (81.9%), meningococcus C (23.5%), varicella (18.4%), pneumococcus (18.6%), papillomavirus (HPV) (5.9%), and rotavirus (1.9%) vaccines. Complete immunization was recorded only in 38/430 (8.8%) children [82]. 

Several factors, including little attention to immunization from pediatric gastroenterologists who follow the patient for IBD and parents’ or patient’s vaccine refusal might explain the poor adherence to the suggested immunization schedules. Among 657 pediatric gastroenterologists from 129 institutions listed in the North American Society for Pediatric Gastroenterology, Hepatology, and Nutrition, only 63.5% and 44.4% were found to assess the immunization status of the patient at the time of diagnosis and before initiating immunosuppressive therapy, respectively [83]. Moreover, at diagnosis, only 9.0% obtained serology to evaluate the protection of children against vaccine preventable diseases. These behaviors were justified with the beliefs that vaccinations were the responsibility of primary care pediatrician, the poor access to immunization records, the lack of a reminder system, and the inability to administer vaccines in the office [83]. Parental refusal was associated with recent movement from another province or country with different immunization schedule, lack of awareness of routine immunization, fear of side effects, and fear of disease activation. Patient’s refusal was mainly due to the needle phobia [81,84]. In the study by Languet et al. [81], poor immunization coverage was ascribed to patient’s refusal in 41% of cases, fear of side effects in 33%, and fear of disease activation in 5%. Importance of awareness of routine immunization seems to be suggested by the evidence that poor compliance to recommendations was greater for the vaccines recently included in the official immunization schedule. A retrospective review of immunization status of 101 Australian children aged 0–18 years showed that 90% (95% CI, 77–97) were up to date with primary routine immunization, whereas only 5% (95% CI, 2–11) had received complete pneumococcal immunization, and 10% (95% CI, 5–17) had evidence of having ever received a seasonal influenza vaccine [85]. Educational programs highlighting the role of vaccines could improve compliance, particularly when they are associated with vaccine access in the center where the children are regularly followed. Huth et al. reported that before any intervention, only 47% of patients received the influenza vaccine [86]. This value rose to 75.0% after education and to 89.5% when education and vaccine access in clinic were combined (*p* = 0.0043). Moreover, a survey of the Italian Group for the Study of Inflammatory Bowel Disease showed that the indication to immunization was given at the diagnosis of the disease by 55.6% of the gastroenterologists, and that efforts carried out by the scientific societies were required to increase the awareness of this relevant topic among physicians [87]. These data showed the importance of educational interventions, including manuscripts, that highlight the risks of vaccine-preventable diseases, as well as the efficacy and the safety of vaccines in patients with IBD. 

### 4.2. Immunogenicity of Vaccine in Children with IBD

If some number of IBD children do not follow official recommendations and are not adequately immunized, a part of them remains poorly protected against vaccine preventable diseases, even when they receive vaccines at proper time [88]. It cannot be precisely defined whether this depends on the immune dysfunctions strictly related to IBD development, on the therapy, or on both these factors. Lower than expected antibody levels can be demonstrated in children who received vaccines both before and after IBD development, and in those with diseases, with or without immunosuppressive therapy. An old study assessing the humoral response of patients with IBDs to tetanus and diphtheria vaccine showed that the in vitro antibody production of specific IgG by B cells and the serum specific IgG titers against both antigens were below the normal values in most cases, regardless of disease activity, type of IBD, and immunosuppressive therapy [89]. In agreement with these findings is the recent evidence that the immunogenicity of the oral cholera vaccine in children with IBD is lower than that observed in healthy ones and the treatment type does not affect the vaccine immunogenicity [90]. Similar conclusions could be drawn by the few studies that reported that the use of immunomodulators or when biologics did not influence the immune response. The very recent study by Baranowska-Nowak et al. is an example in this regard [91]. These authors showed that the rate of seroconversion to HBV in a group of 157 IBD patients with a median age of 14.5 years had no relationship with age (*p* = 0.3), sex (*p* = 0.7), or IBD type (*p* = 0.9), and was totally independent of therapy, including immunosuppressive drugs, biologicals, and combinations. Higher rates of protective anti-HBs levels (≥10 mIU/mL) were observed in patients treated with 5-ASA, immunomodulators without and with biologicals, but the differences were not statistically significant (*p* = 0.4, 0.7, and 0.9, respectively). Finally, it was evidenced that there exists abnormal in vitro cytokine production by peripheral blood mononuclear cells from IBD patients not receiving immunosuppressive therapy or biologics, even when these subjects could be considered in remission, according to disease activity index [92,93]. All these findings seem to suggest that IBDs can induce a reduced vaccine immune response, leading to some children having an increased risk of vaccine-preventable disease. 

In a cross-sectional study carried out in Canada enrolling children aged about 15 years with IBD who had received vaccines at a younger age according to the national immunization schedule, found that, despite a very high vaccine coverage (93.5% for MMR, 95.6% for DTP/polio/Hib, 75.8% for HBV, 93.5% for varicella), seroprotection could be evidenced only in 65.8% of subjects for measles, 60.5% for mumps, 79.1% for rubella, 79.5% for diphtheria, 80.8% for tetanus, 70.5% for varicella, and 62.8% for HBV [94]. For all studied vaccines, values were significantly lower than that detected in healthy subjects, after a similar period of time since vaccine administration. Antibody against varicella could be detected in 95.5% and 95.3% of healthy children who received vaccines 5 and 9 years before [95]. Healthy preadolescents immunized with HB vaccine were shown to have serologic protection in 88.2%, 86.4%, and 76.7% of cases at 5, 10, and 15 years postvaccination, respectively [96]. Measles serologic protection was evidenced in >95% of children aged 7–18 years and >98% of young adults (19–25 years old) [97]. As older age at IBD diagnosis was associated with greater serologic protection, these findings seem to indicate that before disease development, immune system functions are not impaired, a longer duration of normal immune function could favor persistent vaccine protection. Support to this hypothesis is given by the evidence that administration of a diphtheria vaccine booster to adolescents with IBD previously vaccinated according to the recommended immunization schedule permitted to achieve an immune response quite similar to that of healthy children, irrespective of immunosuppressive treatment [98]. In this regard, it would be interesting to understand the persistence of protection of COVID-19 vaccines after the ongoing vaccination campaign. On the other hand, studies that prospectively evaluated immune response to vaccines do not solve the problem definitively. No data in this regard are available regarding DTaP, Hib, and MMR. Moreover, results of studies regarding other vaccines vary significantly according to the vaccine, the time of vaccine administration relative to the time of disease development, and the presence and type of immunosuppressive drugs or biologics for therapy. Details in this regard are reported in the following paragraphs. 

#### 4.2.1. Hepatitis A Vaccine (HAV) 

Most data collected in prospective studies seem to indicate that children with IDBs given HAV, generally have an immune response quite like that usually evidenced in healthy age- and sex-matched subjects without any significant adverse event and exacerbation of disease activity. Seroconversion after the first and the second dose of HAV were generally demonstrated in all or almost all enrolled, previously unimmunized cases, without difference between patients and controls, and independent of the use of immunosuppressive therapy or biologics [99,100]. However, in some cases, subjects receiving immunosuppressive therapy showed a lower seroconversion rate and lower antibody geometric mean titers (GMT), suggesting the risk of poor long-term protection. In a study comparing 66 children with IBD and 68 healthy controls with similar mean age (13.6 ± 0.40 vs. 12.41 ± 0.41 years) and similar distance between HAV doses (9.51 ± 0.29 vs. 8.68 ± 0.28 years), rate of seroconversion after the second dose was 97% in patients and 100% in healthy controls (*p* = 0.2407) [101]. However, treatment with CS but not with AZA significantly influenced the seroconversion rate. In children receiving CS, achievement of seroconversion 4 weeks after the second dose of vaccine was reduced more than 7-fold (*p* = 0.0332; OR, 0.132), whereas no effect was seen in children given AZA or 6-MP. However, difference in seroconversion rate was no longer statistically significant after 12 weeks from the second HAV dose. GMTs against HA were statistically significantly lower in the IBD group than in the control group, after the first and the second dose (*p* < 0.001 for both time-points). Four weeks after the second dose, children receiving CS were found to have more than 3-fold lower GMTs than patients that did not receive CS (44.3 mIU/mL and 149.4 mIU/mL, respectively; *p* = 0.0145). GMTs of children receiving AZA/6-MP were also significantly reduced. Compared to other types of treatment, GMT measured 12 weeks after the second dose of vaccine were 137.3 mIU/mL, as compared to 227.1 mIU/mL (*p* = 0.0475). 

#### 4.2.2. Hepatitis B Vaccine

Reduced immune response to HBV of children with IBD, compared to healthy controls was reported by Urganci and Kalyoncu [99]. These authors administered the HBV to 47 patients and 50 controls, all susceptible to HB infection. Among patients, 13 were receiving CTs and in 8, AZT was added because of CS dependency. Seroprotection (anti-HBs titers 10 mIU/mL) 1 month after the second dose of the primary vaccination was achieved in 70.2% of patients, and 90% of controls achieved seroprotection (95% CI, 0.71–0.87; *p* = 0.02). No correlation between initial vaccine response and the treatment was established. Administration of a booster HBV dose allowed seroprotective titers in 85.1% of patients and 96% of controls (95% CI, 0.83–0.95; *p* = 0.08), even in this case, without correlation with the administered treatment (*p* > 0.05) [99]. 

Immunization was safe as no variations in disease activity or medication use was evidenced. Evaluation of the immune response to HBV of a group of 87 nonimmune children aged 17.9 ± 4.0 years that were receiving infliximab, showed that 49/87 (56%) of them developed an adequate immune response and became immune with a mean concentration of anti-HBs levels of 295.6 ± 350.6 mIU/mL [100]. Infliximab dose, frequency, duration, and the concurrent use of immunomodulators were not associated with no response. Administration of a booster dose to 34 non-responders led to an anamnestic response in 26/34 (76%). Interestingly, non-responders received more frequent doses of infliximab (every 5.9 ± 1.2 weeks vs. every 7.1 ± 1.8 weeks; *p* = 0.01). 

#### 4.2.3. Varicella Vaccine

Despite potentially being at risk, as VZV is based on a live virus, immunization with VZV was found to be safe and effective in some groups of children with reduced immune system efficiency, including those with IBDs. In 6 children with IBD, who had received the primary VZV dose before the diagnosis of IBD and the booster dose after diagnosis, immune response was good in 5 cases and none had relevant clinical problems or worsening of the disease [102]. A prospective, multicenter observational study in which a total of 29 VZV vaccinations were performed in 15 seronegative patients aged 3–16 years, revealed that immunization was well-tolerated and safe, even in subjects with high-intensity immunosuppression [103]. The VZV-IgG-concentration increased significantly (*p* = 0.018) after vaccination, although in few patients, a third dose was needed to achieve protective levels.

#### 4.2.4. Influenza Vaccines

IBD patients have an increased influenza risk, as compared to those without IBD (OR 1.54; 95% CI 1.49–1.63). Moreover, they are more frequently hospitalized (5.4% vs. 1.85%; *p* < 0.001) [103]. For these reasons, it is recommended that they are annually vaccinated with influenza vaccines [103]. Studies in children with IBDs immunized with the inactivated influenza vaccines showed that this prophylactic measure was safe and well-tolerated and did not affect disease activity [104,105,106]. However, immunogenicity was generally shown to be slightly different from that of healthy controls and could vary according to the type of therapy in place at the time of vaccination. Although in some cases, response to influenza A virus was impaired, generally, IBD children had a poor response to influenza B viruses. Both seroconversion rate and achievement of seroprotective antibody concentrations against these vaccine component was frequently found to be lower in IBD patients than in healthy controls [105,106,107]. Moreover, use of immunosuppressive drugs with or without biologics could significantly further reduce response to influenza B virus vaccines’ component. Mamula et al. showed that children with IBD were less likely to achieve serologic protection against the B/Hong Kong/330/2001 component of the 2002–2004 influenza vaccine, as compared to healthy controls (64% versus 90%; *p* = 0.0125) [105]. Moreover, patients treated with immunomodulatory drugs (CS, 6-MP, or MTX) or biologics (IFX) had a lower response to the A/H1N1 virus [104]. Lu et al. showed that only 39% of children with IBD developed serologic protection against the B/Malaysia/2506/2004 component of the 2007 influenza vaccine, as compared to 96% and 88% for A/H1N1 and A/H3N2 [106]. Similar findings were reported by deBruyn et al., who conducted a study enrolling 60 children with IBD and showed that the proportion of patients with an appropriate immunogenic response to the two influenza A subtypes was quite similar to that of the healthy controls (70% for H3N2, 72% for H1N1) [107]. On the contrary, seroconversion rate to the influenza B component was only 53% in children. Moreover, although protective antibody titers were achieved in a high proportion of children with IBD (95% for H3N2, 98% for H1N1, 85% for influenza B), immunosuppressed children were less likely to achieve serologic protection, as compared to otherwise healthy children (79% versus 100%; *p* = 0.02) [105]. Recently, some authors stated that high-dose (HD) influenza vaccine could be a preferred option for immunocompromised patients [107,108]. Results of the study by Caldera et al. [109] seem to confirm this statement. These authors showed that patients with IBD receiving the HD influenza vaccine had a significantly higher H3N2 postimmunization antibodies, as compared to those who received the standard influenza vaccine (160 [interquartile range 80 to 320] vs. 80 [interquartile range 40 to 160]; *p* = 0.003), although the H1N1 postimmunization levels were not significantly different (*p* = 0.18). Administration of vedolizumab did not influence antibody response. However, larger randomized controlled studies are needed to validate the relative immunogenicity and safety of enhanced vaccines in immunocompromised individuals, including those with IBD.

#### 4.2.5. Pneumococcal Vaccines

Response to the 13-valent pneumococcal conjugate vaccine was tested in 178 children aged 5 to 18 years, with no history of pneumococcal immunization (122 with IBD and 56 controls) [110]. The study showed that the number of cases with adequate response (post-vaccination titer ≥0.35 μg/mL to all 13 serotypes) was slightly higher in healthy controls than in patients, although the difference was not statistically significant (90.4% vs. 96.5%; *p* = 0.5281). Among patients, those treated with immunosuppressive therapy had higher geometric mean titers (GMT) than those without (*p* = 0.0369) [110]. Safety and tolerability of both pneumococcal vaccines was very good, not different from those reported for healthy children. No variation in disease activity was evidenced. 

Immunogenicity of the 23-valent polysaccharide pneumococcal vaccine (PPV23) was studied in a group of 18 patients with IBD and in 20 healthy individuals, in all cases without previous pneumococcal immunization [111]. Mean age of patients at study entry was 10.7 ± 4.2 years, whereas mean age at the time of onset of disease was 7.6 ± 3.6 years. Among them, 15 were receiving immunosuppressive drugs. The mean increased level of total IgG after vaccination was significantly lower in IBD patients than in controls (82.9 ± 32.5 µg/mL vs. 219.8 ± 59.0 µg/mL; *p* = 0.001) [111]. Ten out of 18 patients (55.5%) were found to be hyporesponsive to the vaccine as they had an increase in specific antibody titers lower than the limit of the 2-tailed 90% probability interval of postimmunization specific IgG of healthy adults. Among hyporesponsive patients, mean values of IgG levels after immunization (*p* = 0.018), absolute increase in the level of anti-pneumococcal IgG (*p* < 0.001) and its fold increase ratio (*p* = 0.001) were significantly lower than in children with normal immune response [111]. 

#### 4.2.6. Human Papillomavirus (HPV) Vaccines

Administration of HPV vaccine to IBD patients evokes an immune response quite similar to that reported in healthy subjects without any variation in tolerability and safety. Jacobson et al. administered 3 doses of the tetravalent HPV vaccine to 37 previously unvaccinated females aged 9–26 years with IBD, who were prescribed maintenance immunosuppressive therapy for at least 30 days prior to enrollment with any immunomodulator or a TNF-α inhibitor [112]. GMTs against the four HPV types were determined and compared to those found in healthy females of similar age. Seropositivity after dose 3 was 100% for types 6, 11, and 16, and 96% for type 18. GMTs in patients were as high or higher in all four HPV types, as compared to controls. No statistically significant difference between drugs was evidenced, with the exception of GMTs for HPV6, which were higher in the cases treated with anti-TNF-α agents. Analysis of antibody persistence revealed that subjects with IBD immunized up to 27 months before seropositivity remained high, although GMT were slightly lower, as is usually observed in healthy subjects. No severe vaccine-related adverse event was reported. No data are available in males with IBD as well as when using the 9-valent HPV vaccine.

#### 4.2.7. COVID-19 Vaccines

Whether children and young adults with IBD with or without immunomodulating biologics or other therapies are at increased risk of severe COVID-19 or post-infectious co-morbidities, including the recently described multisystem inflammatory syndrome of children [113], is not known. Moreover, the immune response of children with IBD with or without treatment to SARS-CoV-2 infection is unknown. Consequently, it is difficult to establish whether children with IBD must be included in the list of subjects who should be vaccinated first, although the general increased risk of infection in these patients strongly supports it. On the other hand, immunogenicity and safety of the presently authorized for emergency use COVID-19 vaccines, in healthy or diseased children, was not thoroughly investigated and no COVID-19 vaccine was studied in pediatric patients with IBD (i.e., those aged <18 years). To address these problems, a study is planned, in which children with IBD living in a region with high incidence of SARS-CoV-2 infection will be monitored over a two-year period to assess disease incidence rate and antibody development and durability to SARS-CoV-2 (NCT04838834). Moreover, a number of studies for evaluating the immune response of children to COVID-19 vaccines are ongoing, and in some cases, preliminary results in healthy children are already reported. The company that produces one of the available mRNA vaccines recently reported in a press release that this vaccine demonstrated 100% efficacy and robust antibody responses in children aged 12–15 years, exceeding those reported in trials of vaccinated 16–25 years old participants in an earlier analysis, and was well-tolerated. Other studies are ongoing in younger healthy children to evaluate safety, immunogenicity, and efficacy, and definitive results are expected in a short time [114]. It seems very likely that these studies could represent the basis for further specific trials aimed at children with IBD, similarly to what already happens for adults with the same clinical problems (NCT04769258, NCT04818892). 

## 5. Strategies for Immunization in Children with Inflammatory Bowel Disease (IBD) 

Taking into account the potential risk of infections, all health authorities agree with the recommendation that children with IBD must follow the same immunization schedule already available for healthy children, with some limitations regarding live attenuated vaccines. All vaccines can be safely administered in patients who are receiving exclusive enteral nutrition, as well as in those who are receiving antimicrobial therapy or mesalazine [115]. In comparison to the review performed by Di Pasquale and Romano, we included studies performed from 2017 to 2020 and entered more details on immunogenicity of the different vaccines. As for other conditions with immunodeficiency, live attenuated vaccines cannot be administered when the child is treated with immunosuppressive therapy [114]. If immunosuppressive therapy must be initiated, the varicella vaccine and MMR vaccine must be given at least 4 weeks before starting immunosuppressive therapy [114]. If treatment is already in progress, live attenuated vaccines could be administered only after suspension of treatment for 3 months, except for CS therapy, for which the suspension can be limited to one month [114]. It is strongly recommended, that at diagnosis, immunization status is verbally or serologically tested, and vaccines are offered in case the schedule was not respected. Determination of antibody titers is suggested particularly when the immunization status is uncertain or when protection must be assessed to decide whether booster doses must be administered [9,112]. In particular, serologic baseline evaluation is recommended for HBV, MMR, and VZV, and vaccines should be offered if the patient is not immune. Moreover, attention must be paid to periodic booster administration, at least of vaccines such as those against pneumococcus, meningococcal, DTP, and influenza, which have a limited duration of efficacy. Finally, vaccination for COVID-19 in IBD patients and their caregivers should be strongly recommended [78,116]. Studies in the pediatric population are ongoing, but vaccination of adults with IBD as well as of caregivers of IBD patients of any age represent a priority. However, as recently collected data seem to indicate that IBD patients have an impaired serological response to the SARS-CoV-2 infection [117], serological testing and virus surveillance should be considered in immunized IBD subjects to detect suboptimal vaccine responses, persistent infection, and viral evolution. If attenuated serological responses following vaccination were observed, then modified immunization strategies should be designed for millions of patients worldwide. 

Patients with IBD who are receiving immunomodulatory therapies do not have an increased risk of vaccination-induced side effects, regardless of the vaccine that will be offered. Instead, a possible suboptimal vaccination response would have to be evaluated and it is essential that patients with IBD are given clear and unambiguous indications [116].

## 6. Conclusions

Despite detailed quantification of the risk of infections in children with IBDs, particularly those that are vaccine preventable, is lacking. Therefore, it seems highly likely that, as reported in adults, children with these diseases might have infections more frequently than age-matched healthy subjects, and that, at least in some cases, these infections might be even more severe. Fortunately, most of these infections could be prevented when recommended schedules of immunization are carefully followed. Table 1 summarizes the main recommendations. In comparison to one of the last reviews on this topic [115], we included studies performed in the last 4 years, including a special focus on COVID-19 vaccines. Vaccines given to children with IBDs generally have adequate safety and immunogenicity. Attention must be paid to an adequate vaccination program, including the monitoring of antibody titres against viruses included in live attenuated vaccines that could be administered only to children without or with mild immune system function impairment, administration of booster doses, as well as vaccination of caregivers. Unfortunately, compliance to these recommendations is generally low and multidisciplinary educational programs to improve vaccination coverage must be planned, in order to protect all children with IBD from vaccine-preventable diseases.

## Figures and Tables

**Table 1:** Vaccine recommendations for children with inflammatory bowel disease (IBD).

Disease Course	Recommendation	Action
At diagnosis	Evaluation of immunization status	Serologic evaluation of antibody titres against HBV, MMR and VZV
		Administration of all the vaccines recommended for patient’s age according to the national schedule as well as vaccination with MMR and VZV vaccines
During follow-up	Periodic booster doses administration	Booster vaccinations against pneumococcus, meningococcus and DTP
	Annual influenza vaccination	Annual administration of quadrivalent influenza vaccine
	COVID-19 vaccine	Administration of COVID-19 vaccine regardless of type of IBD therapy
	Vaccination of caregivers	Adequate vaccination status
		Annual influenza vaccination
		Administration of COVID-19 vaccine

DTP, diphtheria, tetanus, pertussis; HBV, hepatitis B; MMR, measles, mumps, rubella; VZV, varicella-zoster virus.
